# Effects of Huang Bai (Phellodendri Cortex) and Three Other Herbs on GnRH and GH Levels in GT1–7 and GH3 Cells

**DOI:** 10.1155/2016/9389028

**Published:** 2016-01-26

**Authors:** Sun Haeng Lee, Sung Chul Kwak, Dong Kwan Kim, Sang Woug Park, Hyun Soo Kim, Young-Sik Kim, Donghun Lee, Ju Won Lee, Chang Gon Lee, Hae Kyung Lee, Sung-Min Cho, Yu Jeong Shin, Jin Yong Lee, Hocheol Kim, Gyu Tae Chang

**Affiliations:** ^1^Department of Clinical Korean Medicine, Graduate School, Kyung Hee University, Seoul 02447, Republic of Korea; ^2^Department of Pediatrics of Korean Medicine, Kyung Hee University Medical Center, Seoul 02447, Republic of Korea; ^3^Korea Institute of Science and Technology for Eastern Medicine (KISTEM), NeuMed Inc., Seoul 02440, Republic of Korea; ^4^Department of Herbal Pharmacology, College of Korean Medicine, Kyung Hee University, Seoul 02447, Republic of Korea; ^5^Department of East-West Medical Science, Graduate School of East-West Medical Science, Kyung Hee University, Yongin 17104, Republic of Korea; ^6^Department of Pediatrics of Korean Medicine, Kyung Hee University Hospital at Gangdong, Seoul 05278, Republic of Korea

## Abstract

The present study was to evaluate the effects of Huang Bai, Zhi Mu, Mai Ya, and Xia Ku Cao on hormone using the GT1–7 and GH3 cells. The GT1–7 and GH3 cell lines were incubated with DW; DMSO; and 30, 100, or 300 *μ*g/mL of one of the four extract solutions in serum-free media for 24 hours. The MTT assay was performed to determine the cytotoxicity of the four herbs. The GT1–7 and GH3 cells were incubated in DW, estradiol (GT1–7 only), or noncytotoxic herb solutions in serum-free medium for 24 hours. A quantitative RT-PCR and western blot were performed to measure the GnRH expression in GT1–7 cells and GH expression in GH3 cells. Huang Bai, Zhi Mu, Xia Ku Cao, and Mai Ya inhibited the GnRH mRNA expression in GT1–7 cells, whereas Huang Bai enhanced GH mRNA expression in GH3 cells. Additionally, Xia Ku Cao inhibited GnRH protein expression in GT1–7 cells and Huang Bai promoted GH protein expression in GH3 cells. The findings suggest that Huang Bai can delay puberty by inhibiting GnRH synthesis in the hypothalamus while also accelerating growth by promoting GH synthesis and secretion in the pituitary.

## 1. Introduction

Some medicinal herbs and prescriptions have been reported to treat precocious puberty (PP). For example, in Taiwan, the* Anemarrhena*,* Phellodendron*, and* Rehmannia* Pill, containing Zhi Mu (Rhizoma Anemarrhenae), Huang Bai (Cortex Phellodendri), Di Huang (Radix Rehmanniae), Shan Zhu Yu (Fructus Corni), Shan Yao (Rhizoma Dioscoreae), Mu Dan Pi (Cortex Moutan), Fu Ling (Poria), and Ze Xie (Rhizoma Alismatis), are the most common prescription for PP (70.62%), whereas Mai Ya (Fructus Hordei Germinatus; 20.11%) and Xia Ku Cao (Spica Prunellae; 14.69%) are the most common herbs that are used in combination with a PP prescription [1]. Additionally, in China, Zhi Mu (10.19%), Di Huang (9.87%), and Huang Bai (8.92%) are the herbs most frequently used for yin deficiency with effulgent fire pattern PP, whereas Chai Hu (Radix Bupleuri; 9.09%), Shao Yao (Radix Paeoniae; 6.82%), and Dang Gui (Radix Angelicae Gigantis; 6.06%) are the herbs most frequently used for depressed liver qi transforming into fire pattern PP [2].

The administration of the Nourishing “Yin” Removing “Fire” herbal mixture (Di Huang, Huang Bai, Zhi Mu, and others) to normal rats reportedly downregulates the expression of excitatory amino acid and gonadotropin-releasing hormone (GnRH) mRNA in the hypothalamus; follicle-stimulating hormone (FSH), luteinizing hormone (LH), and GH mRNA in the hypophysis; and insulin-like growth factor- (IGF-) I mRNA in the metaphysic, while upregulating the expression of inhibitory amino acid, neuropeptide Y (NPY), *β*-endorphin, and somatostatin mRNA in the hypothalamus [3, 4]. Additionally, in a rat model of PP, this herbal mixture was shown to delay vaginal opening and to downregulate serum estradiol levels as well as the expression of Kiss-1 and GnRH mRNA in the hypothalamus [5, 6]. The Zhizao granule, which contains Xia Ku Cao, Zhi Zi (Fructus Gardeniae), Chai Hu, Shao Yao, Gou Qi Zi (Fructus Lycii), Zhe Bei Mu (Bulbus Fritillariae), and Huang Qin (Radix Scutellariae), reduces serum FSH and LH levels in PP rats [7]. Similarly, a mixture containing Di Huang, Gui Ban (Plastrum Testudinis), Shan Yao, Shan Zhu Yu, Nu Zhen Zi (Fructus Ligustri Lucidi), Fu Ling, Ze Xie, Mu Dan Pi, and Zhi Mu reduces the ovarian index, uterine index, and ovarian corpus luteum occurrence rate [8].

However, single herbs that are used for the treatment of PP are rarely analyzed. Zi Cao (Radix Lithospermi) has been shown to independently reduce serum FSH, LH, and estradiol levels in rats [9] and to diminish vaginal opening, reduce ovary and uterus weights, and lower serum FSH, LH, and estradiol levels in mice [10]. Furthermore, this herb decreases uterine thickness and reduces serum FSH and LH levels while increasing the height of the femur growth plate in rabbits [10].

In the present study, the most frequently used and effective herbs for the treatment of PP, including Huang Bai, Zhi Mu, Xia Ku Cao, and Mai Ya, were assessed to determine their effect on inhibiting maturation and promoting growth by mRNA and protein analyses of GnRH and GH.

## 2. Materials and Methods

### 2.1. Cell Cultures

GT1–7 cells are from a mouse hypothalamic tumor cell line and release GnRH [11]. The GT1–7 cells used in the present study were offered by Professor Kyungjin Kim from Seoul National University (Seoul, Republic of Korea), and their use was approved by Dr. Pamela L. Mellon from University of California (San Diego, California, USA). GH3 cells are from a rat pituitary tumor cell line and release GH. The GH3 cells used in the present study were provided by the Korean Cell Line Bank (Seoul, Republic of Korea). The GT1–7 cells were cultured in Dulbecco's Modified Eagle's Medium (DMEM, Gibco, Carlsbad, California, USA) and supplemented with 10% fetal bovine serum (FBS, Gibco) and 1% pen/strep antibiotics (Gibco). The GH3 cells were cultured in DMEM with Nutrient Mixture F-12 (DMEM/F-12, Sigma-Aldrich, St. Louis, Missouri, USA) supplemented with 10% FBS (Gibco) and 1% pen/strep antibiotics (Gibco). The cells were plated in 150 mm cell culture dishes and grown in an incubator at 5% CO_2_ and 37°C.

### 2.2. Preparation of the Plant Extract

The cortex of* Phellodendron amurense* (Huang Bai) and the rhizome of* Anemarrhena asphodeloides* (Zhi Mu) were imported from China, whereas the spica of* Prunella vulgaris* (Xia Ku Cao) and the fruits of* Hordeum vulgare* (Mai Ya) were collected in Korea (Kyung Hee Herb Pharm., Seoul, Republic of Korea). Each dried plant was ground to a size <5 mm, and 30 g of each plant particle was extracted with 300 mL of distilled water (DW) at 100°C for 3 hours using a reflux heater (Changshin Science, Seoul, Republic of Korea). The extracted fluid was filtrated with filter paper (Hyundai Micro Co., Seoul, Republic of Korea), and the remaining fluid was evaporated to <150 mL with a rotary evaporator (Sunileyela Co., Gyeonggi, Republic of Korea) and lyophilized with a freeze-dryer (Operon*™*, Seoul, Republic of Korea). The powders were stored at −20°C.

The yields of the freeze-dried Huang Bai, Zhi Mu, Xia Ku Cao, and Mai Ya were approximately 10.40%, 38.07%, 6.60%, and 10.40%, respectively. The Mai Ya extract was not fully dissolved in distilled water; therefore, the Mai Ya extract was dissolved in dimethyl sulfoxide (DMSO; Sigma-Aldrich, D8418), whereas the other three plant extracts were dissolved in DW.

### 2.3. MTT Assay

To assess cytotoxicity, the GT1–7 cells (1 × 10^4^ cells/well) [12] and the GH3 cells (2 × 10^4^ cells/well) [13] were seeded in 96-well cell culture plates and then grown in an incubator for 24 hours. The GT1–7 and GH3 cells were incubated with either DW, DMSO, or 30, 100, or 300 *μ*g/mL of each plant extract solution in serum-free media (SFM) for 24 hours. Then, the treatment solutions were replaced with 100 *μ*L of a 3-(4,5-dimethylthiazol-2-yl)-2,5-diphenyltetrazolium bromide (MTT) solution (Sigma-Aldrich, M5655). Following an incubation period of 1 hour, 100 *μ*L of DMSO was added to the plates and they were shaken for 10 minutes. The optical densities were measured at 570 nm.

### 2.4. RT-PCR

To assess the quantity of messenger ribonucleic acid (mRNA), the GT1–7 cells (5 × 10^5^ cells/well) [14] and the GH3 cells (1 × 10^6^ cells/well) [15] were seeded in 60 mm cell culture dishes with SFM and incubated overnight. The GT1–7 cells were incubated with DW, 10 nM estradiol (Sigma-Aldrich, E2758) [12], or noncytotoxic concentrations of each plant extract solution in SFM for 24 hours, whereas GH3 cells were incubated with DW and noncytotoxic concentrations of the plant extract solutions in SFM for 24 hours.

The total quantities of ribonucleic acid (RNA) from these two cell lines were isolated using the QIAzol® reagent (Qiagen, Venlo, Netherlands) and first-strand complementary deoxyribonucleic acid (cDNA) was synthesized using 1 *μ*g of RNA as a template in a SimpliAmp*™* Thermal Cycler (Applied Biosystems, Waltham, Massachusetts, USA) with a High Capacity cDNA Reverse Transcription Kit (Applied Biosystems) according to the manufacturer's protocols. A quantitative reverse transcription polymerase chain reaction (qRT-PCR) analysis was performed with the cDNA and primers (Table 1) using SYBR® Green Real-Time PCR Master Mix (Applied Biosystems) on a StepOnePlus*™* Real-Time PCR System (Applied Biosystems). Each qRT-PCR was performed in triplicate.

### 2.5. Western Blot

To assess the quantity of protein level, the GT1–7 cells (5 × 10^5^ cells/well) [14] and the GH3 cells (1 × 10^6^ cells/well) [15] were seeded in 60 mm cell culture dishes with SFM and incubated overnight. The GT1–7 cells and the GH3 cells were incubated with DW or noncytotoxic concentrations of each plant extract solution in SFM for 24 hours.

The cells were lysed in a lysis buffer containing 50 mM HEPES (pH 7.5), 150 mM NaCl, 10% glycerol, 1% Triton X-100, 1 mM PMSF, 1 mM EGTA, 1.5 mM MgCl_2_·6H_2_O, 1 mM sodium orthovanadate, and 100 mM sodium fluoride. Protein content was measured using a Bio-Rad colorimetric protein assay kit (Bio-Rad). Protein of 30 *μ*g was separated on SDS-polyacrylamide gels and transferred onto a nitrocellulose membrane. Goat GH antibody (1 : 1000, Santa Cruz Biotech, California, USA), rabbit GnRH antibody (1 : 1000, Santa Cruz Biotech, California, USA), and mouse anti-*β*-actin antibody (1 : 5000, Santa Cruz Biotech, California, USA) were used as primary antibodies. Horseradish peroxidase-conjugated anti-goat antibody for GH (1 : 1000, Millipore, Darmstadt, Germany) was used as secondary antibody. Horseradish peroxidase-conjugated anti-rabbit antibody for GnRH (1 : 1000, Millipore) was used as secondary antibody. Horseradish peroxidase-conjugated anti-mouse antibody for *β*-actin (1 : 1000, Santa Cruz) was used as secondary antibody. Band detection was performed using the enhanced chemiluminescence (ECL) detection system (Amersham Pharmacia Biotech GmbH, Freiburg, Germany). To compare relative expression of proteins, detected bands were calculated densitometrically using Image J (National Institutes of Health, Bethesda, Maryland, USA). Immunoreactive band optical density using GnRH and GH antibody was divided by optical density using *β*-actin antibody.

### 2.6. Statistical Analysis

MTT assay and RT-PCR data were expressed as mean ± standard deviation and western blot data were expressed as mean ± standard error. Data were analyzed with Student's *t*-tests (GraphPad Software Inc., La Jolla, California, USA). A *P* value <0.05 was considered to indicate statistical significance.

## 3. Results

### 3.1. Cytotoxicity

**Figure 1:**
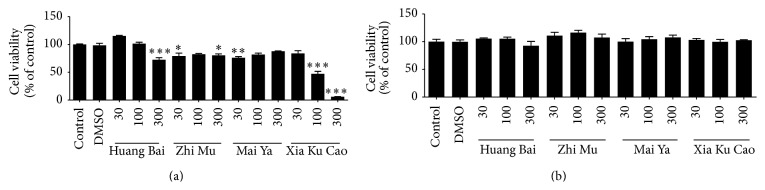
Cell viability of GT1–7 (a) and GH3 (b) cells was analyzed as reduction of MTT to formazan. All results were expressed as the percentage of optical density of control (distilled water treated cells). The results ran in triplicate and expressed as mean ± standard deviation. Statistical analysis of Huang Bai, Zhi Mu, and Xia Ku Cao was compared to control, and that of Mai Ya was compared to DMSO: ^*∗*^ *P* < 0.05, ^*∗∗*^ *P* < 0.01, and ^*∗∗∗*^ *P* < 0.001.

The cell viabilities of the GT1–7 and GH3 cell lines were compared with those of a DW group (Huang Bai, Zhi Mu, and Xia Ku Cao) or a DMSO group (Mai Ya) (Figure 1). The maximum noncytotoxic treatment concentrations of Huang Bai, Zhi Mu, Mai Ya, and Xia Ku Cao in GT1–7 were 30 *μ*g/mL, 100 *μ*g/mL, 100 *μ*g/mL, and 300 *μ*g/mL, respectively. There was only a slight difference between the 100 and 300 *μ*g/mL concentrations of Zhi Mu; therefore, the final treatment concentrations for the extracts in the GT1–7 cells were 30 *μ*g/mL, 300 *μ*g/mL, 100 *μ*g/mL, and 300 *μ*g/mL, respectively. The maximum noncytotoxic treatment concentrations of Huang Bai, Zhi Mu, Mai Ya, and Xia Ku Cao in the GH3 cells were all 300 *μ*g/mL; therefore, the final treatment concentration for all plant extracts in GH3 cells was 300 *μ*g/mL.

### 3.2. GnRH and GH mRNA Expression

**Figure 2:**
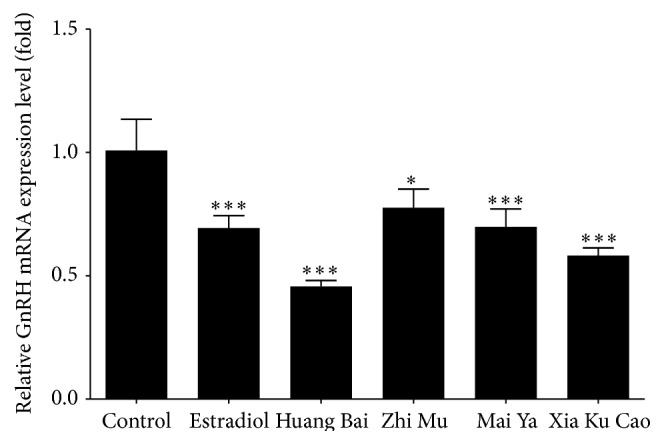
GT1–7 cells were treated with either distilled water (control), 10 nM of estradiol, 100 *μ*g/mL of Huang Bai, 300 *μ*g/mL of Zhi Mu, 300 *μ*g/mL of Mai Ya, or 30 *μ*g/mL of Xia Ku Cao for 24 hours. Total RNAs were extracted and quantitative RT-PCR was performed with GnRH mRNA amplifying primers. The results ran in triplicate and expressed as mean ± standard deviation. Statistical analysis: ^*∗*^ *P* < 0.05, ^*∗∗∗*^ *P* < 0.001 as compared to control.

**Figure 3:**
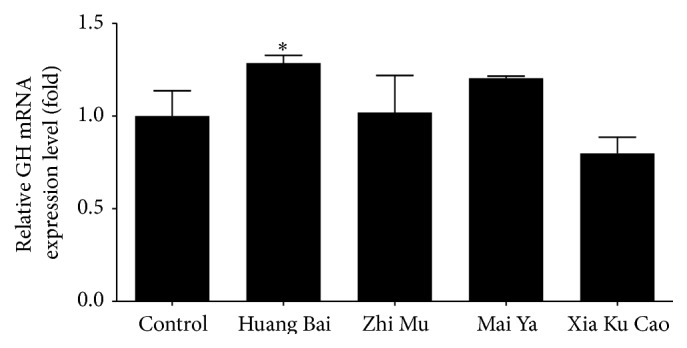
GH3 cells were treated with either distilled water (control), 300 *μ*g/mL of Huang Bai, 300 *μ*g/mL of Zhi Mu, 300 *μ*g/mL of Mai Ya, or 300 *μ*g/mL of Xia Ku Cao for 24 hours. Total RNAs were extracted and quantitative RT-PCR was performed with GH mRNA amplifying primers. The results ran in triplicate and expressed as mean ± standard deviation. Statistical analysis: ^*∗*^ *P* < 0.05 as compared to control.

The expression of GnRH mRNA in the GT1–7 cells and expression of GH mRNA in the GH3 cells were compared with those in a DW group (Figures 2 and 3). The expression of GnRH mRNA changed by 69%, 46%, 78%, 70%, and 58% following treatment with estradiol, Huang Bai, Zhi Mu, Mai Ya, and Xia Ku Cao, respectively. The expression of GH mRNA changed by 129%, 102%, 120%, and 80% following treatment with Huang Bai, Zhi Mu, Mai Ya, and Xia Ku Cao, respectively. Four herbs inhibited GnRH mRNA expression in GT1–7 cells; however, Huang Bai promoted GH mRNA expression in GH3 cells.

### 3.3. GnRH and GH Protein Expression

**Figure 4:**
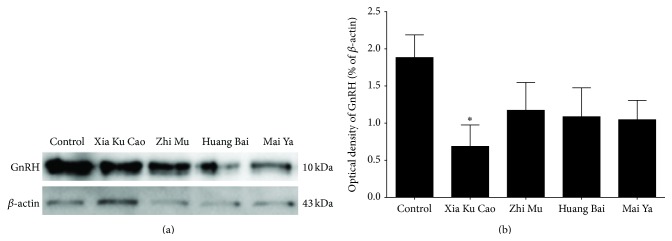
(a) GT1–7 cells were precultured for 24 hours in serum-free media and cultured with distilled water (control), 30 *μ*g/mL of Xia Ku Cao, 300 *μ*g/mL of Zhi Mu, 100 *μ*g/mL of Huang Bai, or 300 *μ*g/mL of Mai Ya. The 24-hour treated cells were lysed and subjected to SDS-PAGE/immunoblotting analysis using anti-GnRH and anti-actin antibodies. Protein bands are representative of three independent experiments. (b) Each band optical density was digitized by NIH Image J. Digitized Analysis of band optical intensity evaluated by adjusted volume. Results are expressed as mean ± standard error of triplicate data. ^*∗*^ *P* < 0.05 as compared with control.

**Figure 5:**
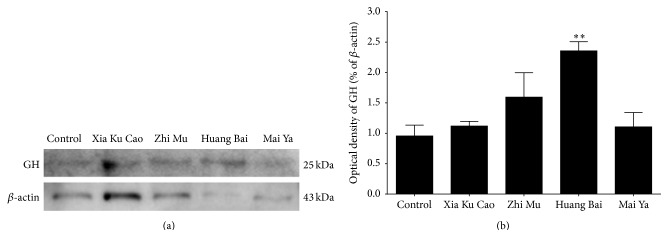
(a) GH3 cells were precultured for 24 hours in serum-free media and cultured with distilled water (control), 30 *μ*g/mL of Xia Ku Cao, 300 *μ*g/mL of Zhi Mu, 100 *μ*g/mL of Huang Bai, or 300 *μ*g/mL of Mai Ya. The 24-hour treated cells were lysed and subjected to SDS-PAGE/immunoblotting analysis using anti-GnRH and anti-actin antibodies. Protein bands are representative of three independent experiments. (b) Each band optical density was digitized by NIH Image J. Digitized Analysis of band optical intensity evaluated by adjusted volume. Results are expressed as mean ± standard error of triplicate data. ^*∗∗*^ *P* < 0.01 as compared with control.

Relative optical densities using GnRH antibody of control, Huang Bai, Zhi Mu, Mai Ya, and Xia Ku Cao were 1.88 ± 0.31, 1.09 ± 0.39, 1.17 ± 0.38, 1.05 ± 0.26, and 0.68 ± 0.29, respectively (Figure 4). Relative optical densities using GH antibody of control, Huang Bai, Zhi Mu, Mai Ya, and Xia Ku Cao were 0.96 ± 0.18, 2.36 ± 0.15, 1.60 ± 0.40, 1.11 ± 0.24, and 1.12 ± 0.08, respectively (Figure 5). Xia Ku Cao inhibited GnRH protein extracts in GT1–7 cells and Huang Bai promoted GH protein extracts in GH3 cells.

## 4. Discussion

The qRT-PCR measurements showed that the water extracts of Huang Bai, Zhi Mu, Mai Ya, and Xia Ku Cao significantly inhibited the GnRH mRNA expression in GT1–7 cells. Of these herbs, Huang Bai showed greater suppressive effect on GnRH mRNA expression than estradiol (*P* < 0.05). Although estradiol is known to bidirectionally regulate the GnRH secreting neuronal function according to the ovulatory cycle in females, it also decreases GnRH gene expression in GT1–7 cells [16]. The GT1–7 cell line is derived from a transgenic mouse hypothalamic tumor and has many of the characteristics of GnRH secreting neurons [11]. Huang Bai and the other three herbs act directly on GnRH neurons which is pivotal for gonadotropin synthesis and release in pituitary. The downregulation of GnRH mRNA mechanism of the four herbs could be established by further receptor signaling study. Different to GnRH mRNA expression, Xia Ku Cao only decreased GnRH protein extracts from GT1–7 cells. Discordance of the results will be studied by further protein synthesis or activation study.

In the present study, the exposure of GH3 cells to the extract of Huang Bai increased the expression of GH mRNA and also increased the expression GH protein. The GH3 cell line is rat pituitary adenoma cell which produces GH and prolactin [17]. Huang Bai also acts directly on GH neurons as well as GnRH neurons. Further studies are necessary in order to decide if increased GH mRNA and protein expression is due to the increase in transcription or stability of the mRNA and protein.

The findings of* in vitro* study suggest that Huang Bai directly inhibits GnRH gene expression in hypothalamus and promotes GH gene and protein expression in pituitary, therefore enhancing possibility of direct actions on GnRH and GH neurosecretory system* in vivo*. Dose- or time-dependent analysis and further* in vivo* studies are needed for clear efficacy of Huang Bai on GnRH and GH system.

Previous studies have demonstrated that the bark of Huang Bai has antidiabetic [18], anti-heat stress [19], anti-inflammatory [20], antimicrobial [21, 22], and neuroprotective [23] effects and that the spike of Xia Ku Cao exhibits antitumor [24, 25] and immunosuppressive [26] activities. Similarly, the rhizome of Zhi Mu possesses antidiabetic, anti-inflammatory, anticoagulatory, antihypertensive, antitumor, antioxidative, antimicrobial, antiviral, antiosteoporosis, anti-skin aging, and neuroprotective [27] effects; additionally, the fruit of Mai Ya exerts antihyperprolactinemia activity [28]. The results of our study newly suggest maturation-inhibiting effect of these four herbs and growth-promoting effect of Huang Bai.

## Figures and Tables

**Table 1:** Specific primers for the qRT-PCR analysis.

Gene		Sequence
GAPDH	F primer	5′-TGGCCTCCAAGGAGTAAGAAAC-3′
	R primer	5′-CAGCAACTGAGGGCCTCTCT-3′

GnRH	F primer	5′-CTACTGCTGACTGTGTGTTTG-3′
	R primer	5′-CATCTTCTTCTGCCTGGCTTC-3′

GH	F primer	5′-ACTCCCTGGCTCCTGACCTT-3′
	R primer	5′-GGATGAGCAGCAGCGAGAA-3′
